# Supplementary material for: A UMLS-based spell checker for natural language processing in vaccine safety
Source: BMC Med Inform Decis Mak. 2007 Feb 12;7:3. doi: 10.1186/1472-6947-7-3 (PMC1805499; doi:10.1186/1472-6947-7-3)
Supplement: Additional file 13 — RAP application directory. Sets up directory for RAP (RDF application for PHP) source code [file 1472-6947-7-3-S13.gz › rap/doc/phpdoc/default/RSS.html]

xml version="1.0" encoding="iso-8859-1"?


Docs For Class RSS


## Class RSS

Description

Description |
Methods (details)

Located in /vocabulary/RSS\_C.php (line 21)

Method Summary

Description |
Methods (details)

void
CHANNEL
()

void
DESCRIPTION
()

void
IMAGE
()

void
ITEM
()

void
ITEMS
()

void
LINK
()

void
NAME
()

void
TEXTINPUT
()

void
TITLE
()

void
URL
()

Methods

Description |
Methods (details)

CHANNEL (line 23)

void

CHANNEL
()

DESCRIPTION (line 71)

void

DESCRIPTION
()

IMAGE (line 29)

void

IMAGE
()

ITEM (line 35)

void

ITEM
()

ITEMS (line 47)

void

ITEMS
()

LINK (line 59)

void

LINK
()

NAME (line 77)

void

NAME
()

TEXTINPUT (line 41)

void

TEXTINPUT
()

TITLE (line 53)

void

TITLE
()

URL (line 65)

void

URL
()

Documentation generated on Fri, 17 Dec 2004 16:18:07 +0100 by phpDocumentor 1.3.0RC3
